# Baxdrostat: A Next-Generation Aldosterone Synthase Inhibitor Offering New Hope in Resistant Hypertension

**DOI:** 10.3390/biom15101439

**Published:** 2025-10-11

**Authors:** Ewelina Młynarska, Witold Czarnik, Natasza Dzieża, Weronika Jędraszak, Gabriela Majchrowicz, Filip Prusinowski, Magdalena Stabrawa, Jacek Rysz, Beata Franczyk

**Affiliations:** 1 Department of Nephrocardiology, Medical University of Lodz, Ul. Zeromskiego 113, 90-549 Lodz, Poland; 2 Department of Nephrology, Hypertension and Family Medicine, Medical University of Lodz, Ul. Zeromskiego 113, 90-549 Lodz, Poland

**Keywords:** arterial hypertension, cardiovascular disease, pathophysiology, molecular mechanisms, complications

## Abstract

Hypertension is a leading global cause of cardiovascular disease and mortality, with resistant hypertension (RH) posing treatment challenges. Aldosterone synthase inhibitors (ASIs) are a novel drug class that reduce blood pressure by lowering aldosterone levels. Baxdrostat is a selective ASI that inhibits the CYP11B2 enzyme, responsible for aldosterone synthesis, without affecting cortisol production. This selectivity minimizes hormonal side effects. Clinical trials have shown that baxdrostat reduces plasma aldosterone in a dose-dependent manner while preserving cortisol levels. In the Phase 2 BrigHTN trial, baxdrostat significantly lowered systolic and diastolic blood pressure in patients with RH, with the 2 mg dose showing the most consistent efficacy. However, in the HALO trial, similar blood pressure reductions were observed in the placebo group, possibly due to improved adherence to background antihypertensive therapy. Baxdrostat has demonstrated a favorable safety profile, with mostly mild adverse effects and no significant impact on kidney function. It is considered safe for use with other medications, including metformin. Ongoing trials are investigating its potential in patients with chronic kidney disease (CKD) and primary hyperaldosteronism (PA). Baxdrostat represents a promising therapeutic option for aldosterone-driven hypertension, especially in patients unresponsive to standard treatments.

## 1. Introduction

Hypertension remains a critical global health concern, contributing substantially to cardiovascular (CV) morbidity and mortality as of 2025 [1]. It is widely recognized as a leading modifiable risk factor for both heart disease and stroke [2]. Despite significant progress in hypertension research and the availability of effective antihypertensive therapies, a considerable proportion of patients fail to achieve adequate BP control, and complications associated with uncontrolled hypertension continue to be prevalent [1]. In 2017, an estimated 55 million deaths were recorded worldwide, with 17.7 million attributed to cardiovascular diseases (CVDs), underscoring the urgent need to monitor the relationship between modifiable risk factors and mortality. This need extends globally and varies according to the economic development level of individual countries, informing the design of effective prevention strategies. Between 2007 and 2017, years of life lost (YLL) due to coronary heart disease (CHD) increased by 17.3%, and due to stroke by 12%, further highlighting hypertension as a major public health challenge [2,3,4]. A disproportionate share of the global CVD burden falls on low- and middle-income countries (LMICs), where limited healthcare infrastructure, financial constraints, and coexisting public health challenges impede effective risk factor control. In these settings, hypertension frequently affects younger individuals, and its sequelae—including stroke and myocardial infarction (MI)—have profound socioeconomic consequences, particularly among working-age populations. These observations reinforce the essential role of primary prevention as the cornerstone of CVD control in LMICs and globally [5].

## 2. Blood Pressure Measurement

BP measurement is a fundamental component in the diagnosis and classification of arterial hypertension (HTA). Currently, three primary measurement methods are distinguished: office blood pressure (OBP) measurement, ambulatory blood pressure monitoring (ABPM), and home blood pressure monitoring (HBPM) [6].

### 2.1. Office Blood Pressure Measurement

OBP measurement can be performed using two methods: auscultatory or oscillometric. The auscultatory method involves manual measurement with a mercury or aneroid sphygmomanometer and a stethoscope, based on identifying Korotkoff sounds over the brachial artery. Manual BP measurement in clinical practice is associated with low accuracy, often leading to overdiagnosis of hypertension and showing poor correlation with ambulatory measurements and target organ damage [7]. An alternative is the oscillometric method, also known as Automated Office Blood Pressure (AOBP), which uses automatic devices that analyze pressure oscillations in the cuff caused by blood flow. The oscillometric technique, used in AOBP, can be conducted both in the presence of medical personnel and without their involvement; however, there is no clear evidence favoring one approach over the other in preventing CV events [8]. AOBP devices typically perform a series of three to six measurements at regular intervals, and the result is reported as an average, which increases the reliability of the measurement and reduces the white coat effect [8,9]. Although this technology has improved accuracy and simplified the measurement process, challenges remain in fully standardizing procedures, which may affect comparability of results across different clinical settings [10].

According to the 2024 ESC/ESH Guidelines, hypertension is diagnosed when office systolic blood pressure (SBP) values are ≥140 mm Hg or diastolic blood pressure (DBP) values are ≥90 mm Hg, provided these results are confirmed by HBPM, ABPM, or repeated office measurements during a follow-up visit. Elevated BP in the office setting is defined as values ranging from 120/70 to <140/90 mm Hg, which require further observation and potential preventive measures [8].

### 2.2. HBPM—Home Blood Pressure Monitoring

HBPM is a technique that is gaining popularity and is increasingly used in clinical practice. It is becoming widely accepted by both patients and healthcare professionals, primarily due to the availability of reliable and user-friendly devices, as well as its greater accuracy compared to office measurements. HBPM reduces the white coat effect [11], allows for the collection of multiple readings over a short period, and better reflects true BP values, thereby enabling more accurate CV risk assessment [12]. In addition, regular use of this method—particularly when combined with medical supervision—supports hypertension management and enhances patient engagement in the treatment process [13].

HBPM is now widely applied in the assessment of BP, with standard practice involving two measurements in the morning and two in the evening over a 7-day period. However, in many cases, reliable diagnostic conclusions can be drawn after just 3 days if the results are consistent [14]. Hypertension is diagnosed when the average home BP is ≥135/85 mm Hg, which corresponds to office values of ≥140/90 mm Hg. Systolic values between 120 and 134 mm Hg or diastolic values between 70 and 84 mm Hg are considered elevated BP in home measurements [8,14].

### 2.3. ABPM—Ambulatory Blood Pressure Monitoring

ABPM enables continuous BP measurement during routine daily activities. This automated method records BP over a 24 h period at regular intervals—typically every 15–30 min during the day (e.g., from 6:00 a.m. to 10:00 p.m.) and every 30–60 min at night (e.g., from 10:00 p.m. to 6:00 a.m.). For an ABPM recording to be considered valid and complete, at least 70% of the scheduled readings must be obtained, including a minimum of 20 valid daytime measurements and at least 7 during the night [15].

The diagnosis of hypertension using ABPM is based on the average BP values obtained over the 24 h monitoring period. Hypertension is diagnosed if the 24 h average BP is ≥130/80 mm Hg. These thresholds may also vary depending on the time of day: a daytime average of ≥135/85 mm Hg or a nighttime average of ≥120/70 mm Hg is also indicative of hypertension [8,15].

## 3. Classification of Elevated Blood Pressure

According to the 2024 ESC/ESH Guidelines for the Management of Arterial Hypertension, OBP measurements are classified into three main categories: non-elevated, elevated, and hypertension. Non-elevated blood pressure—replacing earlier terms such as “optimal” or “normal”—refers to values below 120/70 mmHg. Elevated BP is defined by office measurements ranging from 120 to 139 mmHg systolic or 70 to 89 mmHg diastolic. Pharmacological treatment is generally not recommended in this group; however, individuals at high CV risk may benefit from individualized non-pharmacological or pharmacological interventions. Hypertension is diagnosed when OBP is ≥140 mmHg systolic or ≥90 mmHg diastolic. This diagnosis should be confirmed by out-of-office measurements (HBPM or ABPM) or by repeated measurements during a follow-up visit. Table 1 presents the threshold values used to identify elevated BP and hypertension depending on the measurement method: office-based, HBPM, or 24 h ABPM [1,8].

**Table 1:** Based on the ESC/ESH 2024 Guidelines, diagnostic thresholds for elevated BP and hypertension were determined, taking into account various measurement methods: in a doctor’s office, in the home environment, and as part of ABPM [1,8].

	Non-Elevated BP	Elevated BP	Hypertension
Office BP (mmHg)	<120/70	120/70–<140/90	≥140/90
Home BP (mmHg)	<120/70	120/70–<135/85	≥135/85
Daytime ABPM (mmHg)	<120/70	120/70–<135/85	≥135/85
24 h ABPM (mmHg)	<115/65	115/65–<130/80	≥130/80

## 4. Complications

### 4.1. Stroke

Stroke is the second leading cause of mortality worldwide and one of the leading causes of disability [16]. The most important risk factor for stroke is hypertension, which was found in 64% of stroke patients [17,18]. Chronic hypertension was responsible for approximately 50% of ischemic strokes and as many as 70% of hemorrhagic strokes [19]. Studies confirm that antihypertensive treatment is an effective method for both primary and secondary prevention of stroke [20]. A 10 mm Hg reduction in systolic BP was associated with a 27–41% reduction in the risk of stroke [21].

### 4.2. Myocardial Infarction

Hypertension is one of the main risk factors for MI, promoting the development of atherosclerosis and unstable atherosclerotic plaques. It occurs in 31–75% of patients with acute MI, particularly in older adults and with comorbidities such as diabetes mellitus (DM) or heart failure (HF). The presence of hypertension worsens the prognosis, increasing the risk of complications and mortality. However, treatment directed at blocking the renin–angiotensin–aldosterone system (RAAS) can provide significant benefits [22,23].

### 4.3. Heart Failure

Hypertension is one of the most important and common risk factors for the development of HF [24]. The Framingham Heart Study found that 91% of patients who developed HF within 20 years had a prior history of hypertension. The mean time from hypertension diagnosis to the development of HF was 14.1 years. Chronic hypertension primarily contributes to persistent left ventricular (LV) pressure overload and increased intravascular volume, leading to changes leading to the onset of HF [25]. Individuals with hypertension had a 71% higher relative risk of developing HF compared to those without this condition. Furthermore, each 20 mmHg increase in SBP and 10 mmHg increase in DBP were associated with a 28% and 12% increased risk of HF, respectively [26]. Effective BP control with available antihypertensive medications plays a key role in preventing both CV events and the development of HF [27]. Studies have shown that a 10 mmHg reduction in SBP is associated with a significant 28% reduction in the risk of HF [28].

### 4.4. Chronic Kidney Disease

Hypertensive nephropathy (HN) is a complication of chronic, poorly controlled hypertension and is the second most common cause of end-stage renal disease (ESRD) [29,30]. It typically develops within 5–10 years of the onset of hypertension [31]. It is estimated that CKD develops in over 20% of patients with hypertension [32]. Typical clinical symptoms include nocturia, proteinuria, and a decrease in the glomerular filtration rate (GFR) [33]. The diagnosis of HN is based on the presence of impaired renal function and/or albuminuria [34]. In the treatment of hypertension associated with nephropathy, a combination of drugs that block the renin–angiotensin system (RAS) (ACE inhibitors or angiotensin II receptor antagonists) with calcium channel blockers (CCBs) or diuretics is recommended, which allows for more effective BP control and protection of renal function [35].

## 5. Treatment

### 5.1. Non-Pharmacological

Non-pharmacological methods also play an important role in the treatment of hypertension, including

- Reducing salt intake;
- Increasing physical activity;
- Losing weight;
- Increasing potassium intake;
- Reducing alcohol consumption;
- Quitting smoking [8].

Excessive sodium intake is one of the main factors contributing to the development of hypertension, and therefore limiting its intake is widely recommended to lower BP [36]. As part of non-pharmacological treatment, patients are advised to limit their daily salt intake to 4–6 g, which corresponds to approximately 1.6–2.4 g of sodium per day [37]. Meta-analyses show that an identical reduction in sodium intake results in a significantly greater reduction in BP in patients with hypertension than in normotensive individuals [38]. A proportional relationship has been observed—the greater the dietary salt restriction, the greater the decrease in BP [39,40]. Regular physical activity complements dietary interventions and is an important element of non-pharmacological treatment of hypertension. Among various forms of physical exercise, aerobic exercise performed systematically demonstrates the greatest effectiveness in reducing both SBP and DBP [41,42]. Another important element of non-pharmacological treatment of hypertension is body weight control. Obesity is a well-documented risk factor for increased SBP and DBP [43]. In most individuals with excess body weight, weight loss is associated with a significant reduction in BP [44]. Studies indicate that weight loss of approximately 5 kg results in an average reduction in SBP by 4.4 mmHg and DBP by 3.6 mmHg [45].

### 5.2. Pharmacological

The primary goal of hypertension treatment is effective BP control, which leads to reduced mortality and a reduced risk of adverse CV events [46]. Hypertension treatment should be initiated when BP exceeds 140 or 90 mmHg, regardless of CVD risk [8]. During treatment initiation, non-pharmacological and pharmacological methods should be combined [47]. In patients with primary hypertension, initial treatment is recommended with one of the three main drug classes: thiazide diuretics, angiotensin-converting enzyme inhibitors (ACEIs) or angiotensin II receptor blockers (ARBs), and CCBs [48]. When initiating hypertension treatment, a low-dose combination of two drugs from the main antihypertensive classes is recommended. This approach lowers BP more effectively than monotherapy while reducing the risk of adverse effects [49]. If dual therapy fails to achieve the desired results, a third drug from the main antihypertensive classes should be added. If the therapeutic goal is not achieved, the doses of all three drugs should be gradually increased to the maximum [8].

## 6. The Role of Aldosterone and Cortisol in Hypertension

### 6.1. Characteristics of Aldosterone

Aldosterone is a mineralocorticoid hormone with a direct physiological effect on targets such as cardiomyocytes expressing mineralocorticoid receptors (MRs) [50]. In large part, it is made in the adrenal cortex by the zona glomerulosa cells using the aldosterone synthase (CYP11B2), and in addition is produced in other tissues in the body, where it acts locally [51]. There are several factors stimulating the synthesis of aldosterone, such as extracellular potassium level, the RAS, and adrenocorticotrophic hormone (ACTH) [52].

**Figure 2:**
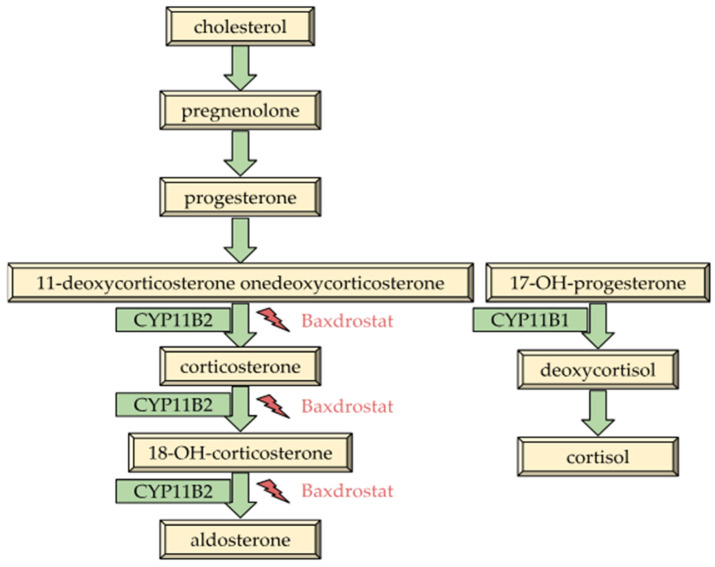
Pathways of aldosterone synthesis [97].

Cholesterol, which is delivered to the inner mitochondrial membrane, is the source of aldosterone biosynthesis (Figure 2. There it is converted by the cytochrome cholesterol side-chain cleavage enzyme (P450scc) into pregnenolone [53]. This precursor is subsequently delivered to the smooth endoplasmic reticulum, where it undergoes enzymatic modifications by 3β-hydroxysteroid dehydrogenase and 21-hydroxylase to form 11-deoxycorticosterone (11DCS). The process then returns to the mitochondria, where 11DCS is sequentially hydroxylated at the 11 and 18 positions and finally oxidized by aldosterone synthase, resulting in the formation of aldosterone [54]. Once synthesized, aldosterone diffuses via the plasma membrane and connects within the cytoplasm with the MR. This activates numerous processes, consequently leading to the transcription of target genes and the production of proteins, including SGK-1 (serum and glucocorticoid-stimulated kinase 1). SGK-1 functions as a key regulatory enzyme that manages the activity of ion transport proteins, including cells in the epithelial sodium channel (ENaC) [55], which initiate water and sodium reabsorption, playing an important role in sodium homeostasis and fluid volume [56].

### 6.2. Mechanisms Leading to Hypertension Related to Aldosterone Activity

#### 6.2.1. Sodium and Water Retention

Aldosterone increases the appearance of ENaC and sodium–potassium ATPase pumps in the main cells of the distal tube, as well as collecting ducts within their plasma membranes. ENaC is responsible for enabling the passive flow of sodium ions, driven by a transepithelial voltage gradient of roughly –50 mV, which in turn is preserved due to the appearance of sodium–potassium ATPase using the ATP to exchange actively intracellular sodium for extracellular potassium. Collectively, these processes enable the reabsorption of sodium from the tubular fluid. Moreover, the penetrability of the collecting duct for water improves, which lets water follow the reabsorbed sodium osmotically into the bloodstream, as a result raising plasma osmolality and promoting water conservation [57]. Consequently, increased water and sodium intake increases circulating blood volume and leads to hypertension.

#### 6.2.2. RAAS Activation

**Figure 1:**
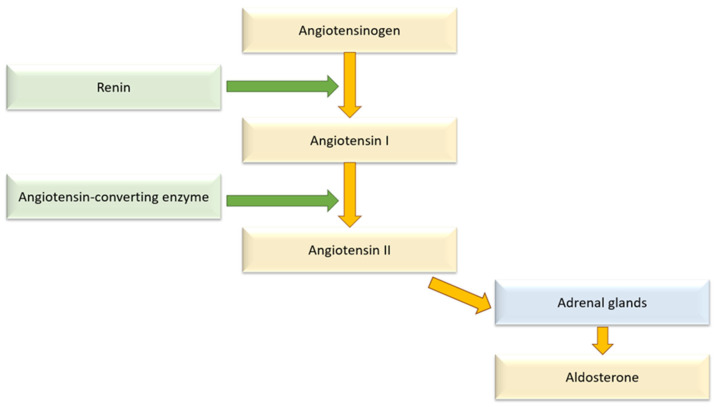
Mechanism of action of the renin–angiotensin–aldosterone system.

Aldosterone is part of the RAAS, which is activated to help restore BP in the event of hypotension by promoting vasoconstriction and the retention of sodium and water; however, its permanent overactivity can cause the development of hypertension. Additionally, renin is released by juxtaglomerular cells located in the kidneys during states of low perfusion and initiates the cascade by changing angiotensinogen into angiotensin I. This factor is then converted into angiotensin II through the action of ACE and then affects the adrenal cortex to release aldosterone as presented in Figure 1. This hormone stimulates increased BP via renal sodium and water reabsorption while promoting potassium excretion; it also acts directly on blood vessels to induce vasoconstriction and triggers the release of arginine vasopressin, a hormone which is a vasopressor factor [58].

#### 6.2.3. Remodeling of Vessels in Cardiovascular System

Binding of aldosterone with MR has been shown to trigger pro-inflammatory mechanisms, mainly by elevating the oxidative stress levels. This form of stress results in the production of free radicals increasing, particularly reactive oxygen species (ROS), which are capable of modifying protein structure and interfering with normal cellular signaling, causing tissue injury and cell death, but also inflammation and fibrosis. Aldosterone contributes directly to this process by increasing ROS formation, particularly affecting vascular tissues and promoting oxidative damage [59], and consequently initiating cardiovascular inflammation and remodeling LV after MI [60] and leading to an increase in left ventricle end-diastolic pressure (LVEDP) [61].

Through its stimulation of ENaC, aldosterone plays a critical role in maintaining sodium homeostasis and regulating BP. Increased ENaC activity, caused by the elevated aldosterone levels, initiates a series of pathological alterations, such as endothelial stiffness, oxidative stress, and inflammation, that together increase the probability of vascular stiffness and aortic structural remodeling, which can generate hypertension [62].

#### 6.2.4. Hypokalemic Nephropathy

Aldosterone is believed to cause hypertension due to its harmful influence on renal function by enhancing the excretion of potassium in urine. Prolonged potassium loss has long been associated with impaired kidney function and lead to hypokalemic nephropathy. This condition is associated with lower medullary perfusion and elevation of vasoactive mediators that promote renal vasoconstriction and disrupt angiogenic processes within the kidney [63].

### 6.3. Characteristics of Cortisol

Cortisol is the glucocorticoid produced in the kidneys by the adrenal cortex, released in the circadian rhythm—with the highest concentration in the morning and gradually declining during the day [64]. Concentration of this hormone is managed locally in the tissues by 11β-hydroxysteroid dehydrogenase (11β-HSD) enzymes and generally by the hypothalamic–pituitary–adrenal (HPA) axis [64]. Cortisol is responsible for many functions, such as reducing the immune response, redirecting energy to organs requiring the energy, such as the brain or muscles, from other organs which are less important for survival [65], and also providing a suitable stress response. Exposure to physical or emotional stress triggers an elevation in cortisol, enabling the body to access energy stores and perform an appropriate response to the challenge [66]. It should be brought into consideration that this hormonal reaction has an advantage in short-term stress, but for longer periods of time it may cause profound physiological and mental health outcomes [67].

Once synthesized, glucocorticoids are secreted into the bloodstream following a sequence of enzymatic reactions occurring within the mitochondria and endoplasmic reticulum. These steps begin with the conversion of cholesterol into pregnenolone by the mitochondrial enzyme cytochrome P450scc. Cholesterol transport into the mitochondria is regulated by the steroidogenic acute regulatory protein (StAR), whose activity is enhanced through phosphorylation. This phosphorylation, along with that of hormone-sensitive lipase (HSL)—which enhances intracellular cholesterol—is triggered by the activation of the protein kinase A (PKA) pathway in response to ACTH. Through this non-genomic signaling cascade, ACTH regulates adrenal steroidogenesis, ultimately driving the production of biologically active glucocorticoids, including cortisol, from cholesterol [68].

### 6.4. Mechanisms Leading to Hypertension Related to Cortisol Activity

#### 6.4.1. Nitric Oxide System

Cortisol is believed to contribute to hypertension by impairing nitric oxide (NO) signaling. It reduces NO availability by inhibiting NO synthase enzymes (iNOS—inducible nitric oxide synthase and eNOS—endothelial nitric oxide synthase), limiting arginine transport via cell membranes and blocking the synthesis of the essential cofactor tetrahydrobiopterin [69]. Animal and human studies [70,71] show that cortisol raises BP while lowering nitrate/nitrite levels in the plasma, independent of changes in arginine or NO synthase inhibitors. Cortisol also weakens the vasodilation of the cholinergic system, suggesting a direct disruption of NO-mediated vascular function [72]. These findings point to NO system dysregulation as a key mechanism in cortisol-induced hypertension.

#### 6.4.2. Erythropoietin

Increased BP is linked to elevated erythropoietin (EPO) levels in individuals with essential hypertension due to the vasoconstrictor action of EPO [73]. Administration of cortisol at a dose of 200 mg/day led to elevation in systolic BP and EPO levels in the plasma, with a clear positive correlation causing a rise in EPO concentration and BP [74]. Nitric oxide resistance has been proposed as a contributing mechanism in EPO-induced hypertension and constant exposure to elevated glucocorticoid levels is known to cause polycythemia [75].

#### 6.4.3. Sodium and Water Retention

Steroid hormones are widely believed to induce hypertension by activating renal type I MRs, promoting the retention of sodium and water as a consequence—a probable cause of cortisol-induced hypertension [76]. However, supporting evidence for this cause-and-effect relationship is limited [77]. In fact, available data suggest a possible dissociation between sodium retention and the hypertensive effects of cortisol [78]. Furthermore, while sodium retention at lower cortisol concentrations may occur via MR activation, higher levels of cortisol appear to dysregulate these receptors, proposing that alternative pathways may mediate sodium retention in such cases.

### 6.5. Adrenal Steroid Modulation in the Management of Hypertension

Pharmacological strategies that modulate adrenal steroid biosynthesis and receptor-mediated signaling have emerged as effective complementary approaches in the management of hypertension, particularly in resistant forms [79,80]. These agents regulate BP by either suppressing the production of adrenal corticosteroids—mainly aldosterone and cortisol—or by antagonizing their effects at specific receptor sites. The therapeutic landscape includes mineralocorticoid receptor antagonists (MRAs), such as spironolactone and eplerenone, which counteract aldosterone’s actions on sodium retention and vascular remodeling [79]. Additionally, adrenal steroidogenesis inhibitors (ASIs) have demonstrated efficacy in reducing corticosteroid output by blocking key enzymatic steps in steroid biosynthesis, offering particular benefit for patients whose hypertension is driven by excessive adrenal hormone production [81]. These emerging agents may enhance BP control while minimizing systemic side effects. Glucocorticoid receptor antagonists also play a role in the management of hypertension in patients with Cushing’s syndrome.

#### 6.5.1. Mineralocorticoid Receptor Antagonists

MRAs, particularly spironolactone and eplerenone, remain cornerstone therapies for resistant hypertension and primary aldosteronism. These agents act by antagonizing aldosterone at its receptor site in the distal nephron, thereby promoting natriuresis, reducing extracellular volume, and lowering systemic BP. While spironolactone is highly effective, its lack of receptor selectivity leads to endocrine-related side effects, including gynecomastia and menstrual irregularities. Eplerenone, with improved receptor specificity, exhibits a more favorable side effect profile but is approximately 25–50% less potent in lowering BP [82,83].

Non-steroidal MRAs represent a significant therapeutic advancement. These agents exhibit high specificity for the MR, minimizing hormonal side effects and the risk of hyperkalemia. Compounds such as finerenone and esaxerenone, both approved for clinical use, demonstrate distinct pharmacokinetic and pharmacodynamic properties compared to steroidal MRAs [84]. For instance, finerenone displays a balanced tissue distribution between the heart and kidneys, and exhibits superior anti-inflammatory and anti-fibrotic activity in preclinical models, particularly at equinatriuretic doses [83].

Although newer agents like finerenone offer a more favorable benefit–risk profile, MRAs remain underutilized in clinical practice, primarily due to concerns regarding hyperkalemia. However, emerging data suggest that non-steroidal MRAs may provide improved cardiorenal outcomes with fewer adverse effects. Continued comparative evaluation of steroidal and non-steroidal MRAs is essential to guide personalized treatment strategies for patients with hypertension and comorbid CV or renal disease [83,84].

#### 6.5.2. Aldosterone Synthase Inhibitors

ASIs represent a novel class of antihypertensive agents that target the final step of aldosterone biosynthesis by selectively inhibiting CYP11B2 in the adrenal zona glomerulosa. This upstream mechanism directly addresses aldosterone-driven hypertension, offering an alternative to MRAs, which act downstream and are often associated with adverse effects such as hyperkalemia and hormonal disturbances due to off-target interactions [84,85].

Osilodrostat, a dual CYP11B1 and CYP11B2 inhibitor, has shown efficacy in patients with Cushing’s syndrome, particularly those with coexisting hypertension and metabolic disturbances. Clinical studies have demonstrated rapid and sustained improvements in both BP and glycemic control in this population [86,87].

Next-generation ASIs, including baxdrostat and lorundrostat, have been engineered for greater selectivity toward CYP11B2, thereby preserving cortisol synthesis and reducing the risk of cortisol-related adverse effects. Early-phase clinical trials report significant reductions in SBP among patients with treatment-resistant or uncontrolled hypertension, especially those with elevated aldosterone levels [88,89].

Although ASIs maintain endogenous cortisol activity and mitigate some of the limitations associated with MR blockade, they may not completely replace MRAs in conditions characterized by ligand-independent MR activation. Nonetheless, ASIs hold therapeutic potential beyond hypertension, particularly in cardiorenal disorders such as HF and CKD [88].

#### 6.5.3. Glucocorticoid Receptor Antagonists

Mifepristone, a glucocorticoid receptor (GR) antagonist, is indicated for the treatment of refractory Cushing’s syndrome with concurrent hypertension, where it improves both BP and glycemic control. However, its clinical use is limited by side effects, including hypokalemia, cortisol withdrawal symptoms, and its antagonism of progesterone receptors, which may result in uterine complications in women. In contrast, relacorilant—a selective GR modulator—has demonstrated favorable safety and efficacy in early clinical trials, improving metabolic parameters and lowering BP in affected individuals [90,91].

## 7. Baxdrostat

### 7.1. Characteristics of Baxdrostat

In recent years, ASIs have emerged as a promising therapeutic approach for managing hypertension by suppressing aldosterone production. These agents offer potential as alternatives to MRAs, particularly in cases of RH. However, earlier ASIs encountered several challenges, including suboptimal effectiveness and adverse side effect profiles. For example, LCI699—one of the first ASIs introduced—demonstrated inferior efficacy when compared to eplerenone. It also displayed reduced selectivity for aldosterone synthase at higher dosages and unintentionally inhibited cortisol synthesis. Interestingly, this cortisol-suppressing effect of LCI699 contributed to the development of Osilodrostat, a medication later approved by the FDA for treating Cushing’s disease. LY3045697, developed subsequently, exhibited improved specificity for aldosterone synthase relative to LCI699. Nevertheless, over prolonged use, its potency was found to be insufficient, requiring higher doses to achieve comparable therapeutic results [92].

Baxdrostat, formerly known as CIN-107 or RO6836191, is a new drug belonging to the selective ASI class [93]. Baxdrostat acts by inhibiting the CYP11B2 enzyme, an aldosterone synthase, which manages the latter stages of aldosterone synthesis as shown in Figure 2 [94]. It has been found to be highly selective for the CYP11B2 enzyme while possessing lower selectivity towards 11b-hydroxylase than other ASI drugs [95,96].

Baxdrostat is a parent compound, and, as its metabolites are present at lower concentrations, it is believed to be primarily responsible for the drug’s action [98]. Yet, the active metabolites have been found to be greatly selective towards aldosterone synthase over 11β-hydroxylase, similarly to their parent compound [99]. Baxdrostat’s main metabolite is CIN-107-M, which is chiral [99]. However, it has been observed that its more potent *R* enantiomer does not form in humans [99]. Given the promising preclinical and early-phase clinical outcomes observed with Baxdrostat, further evaluation at steady state was warranted to determine whether its effects persist after repeated dosing. This need arises from observations with two prior aldosterone synthase inhibitors, which showed diminished efficacy over time. For instance, in multiple-dose trials, LCI699 exhibited reduced selectivity for aldosterone synthase relative to 11β-hydroxylase. This led to notable increases in 11-deoxycorticosterone, accumulation of 11-deoxycortisol, and impaired cortisol production in response to ACTH—suggesting unintended inhibition of cortisol synthesis by LCI699. Similarly, LY3045697 demonstrated reduced potency with chronic dosing. This decline could not be attributed to elevated precursor levels but was hypothesized to result from upregulation of aldosterone synthase activity over time [98].

### 7.2. Pharmacokinetics and Efficacy of Baxdrostat

Baxdrostat has been shown to block synthesis of aldosterone through a dose-dependent reduction in plasma aldosterone and without influencing ACTH-stimulated plasma cortisol levels, both in a preclinical monkey model and in a human study [99,100]. These findings have been highly favorable, since inhibition of cortisol could possibly cause inadequate metabolism, a weakened immune system, and even increased mortality rates [101,102]. Studies have found baxdrostat to have a half-life of approximately 29 h, thus supporting once-daily dosing [98]. Moreover, baxdrostat’s high selectivity for aldosterone synthase has been confirmed, as an increase in the precursor levels 11-deoxycorticosterone and 11-deoxycortisol has only been observed at a dose of ≥90 mg [100].

Phase 1, a placebo-controlled trial by Freeman et al. [98], has assessed baxdrostat’s safety and tolerability at different dosages (0.25, 0.5, 1, and 2 mg once daily) in healthy individuals with a normal- or low-salt diet. The low-salt diet has been used to induce aldosterone production [98]. The trial has found baxdrostat to stimulate a dose-dependent decline in plasma aldosterone levels at doses ≥ 1.5 mg under both diets [98].

Phase 2, a multicenter, placebo-controlled BrigHTN trial by Freeman et al. [94], has studied the efficacy of baxdrostat in 275 patients with RH. Patients with a BP of ≥130/80 mmHg despite therapy with ≥3 drugs at adequate doses, including one diuretic, and an eGFR (estimated glomerular filtration rate) of ≥45 mL/min/1.73 m^2^ were administered placebo or 0.5 mg, 1 mg, or 2 mg of baxdrostat once daily [94]. After 12 weeks, statistically significant reductions in SBP of −20.3 mm Hg, −17.5 mm Hg, −12.1 mm Hg, and −9.4 mm Hg were observed in the 2 mg, 1 mg, 0.5 mg, and placebo groups, respectively [94]. Moreover, baxdrostat has been confirmed to be an effective inhibitor of aldosterone synthase, as it led to a dose-dependent reduction in plasma and urine aldosterone levels [94]. The group receiving 2 mg baxdrostat daily achieved the trial’s secondary endpoint with a reduction in DBP of −5.2 mmHg [94]. In that group, roughly 46% of patients accomplished BP control, understood as SBP  <  130 mmHg [94].

However, the HALO trial, which studied the efficacy of baxdrostat in 249 patients with uncontrolled hypertension, has found no significant difference in OBP after 8 weeks of baxdrostat therapy as compared to the placebo group [103]. Surprisingly, a reduction in SBP has been observed in the placebo group, seemingly because of better adherence to background antihypertensive therapy [103]. Yet, it has been found that patients with therapeutic plasma baxdrostat levels, supposedly due to better compliance with baxdrostat therapy, did achieve a significant decrease in office SBP [103]. The results from the BrigHTN and the HALO trials are shown in Table 2.

**Table 2:** Results from the BrigHTN and the HALO trials.

The BrigHTN trial [95]			
	dose	SBP [mmHg]	DBP [mmHg]
placebo		−9.4	−9.2
baxdrostat	0.5 mg	−12.1	−8.6
	1 mg	−17.5	−11.8
	2 mg	−20.3	−14.3
the HALO trial [104]			
	dose	SBP [mmHg]	DBP [mmHg]
placebo		−16.6	−5.9
baxdrostat	0.5 mg	−17.0	−5.8
	1 mg	−16.0	−5.0
	2 mg	−19.8	−5.4

Another phase 1 study by Freeman et al. [104] has assessed the safety of baxdrostat in patients with varying degrees of renal function. Participants were divided into a control group (eGFR ≥ 60 mL/min), moderate to severe renal impairment group (eGFR 15–59 mL/min), and kidney failure group (eGFR <15 mL/min) and were administered a dose of 10 mg baxdrostat daily [104]. Baxdrostat has been found to be safe in patients with reduced renal function [104]. There has been only one mild adverse effect observed—diarrhea [104]. The study has found that renal dysfunction did not significantly impact systemic exposure or clearance of baxdrostat, thus suggesting that dose adjustment in patients with renal impairment is unnecessary [104].

Currently, there are many ongoing trials investigating baxdrostat’s efficacy and role in therapy [105]. These include the BaxHTN trial (NCT 06034743), a phase 3, multicenter, randomized, double-blinded, placebo-controlled study that has already investigated the effectiveness and safety of baxdrostat at a dose of 1 mg or 2 mg in 720 patients with uncontrolled hypertension on at least two antihypertensive drugs [104]. The phase 2 trial NCT05432167 will evaluate baxdrostat’s role in treating patients with uncontrolled hypertension and mild to moderate CKD [82]. Moreover, the phase 2 trial NCT04605549 will study the effect of baxdrostat on participants with primary hyperaldosteronism [106].

Baxdrostat is a novel drug that so far has shown a very promising effect in hypertension treatment [106]. Overall, selective CYP11B2 inhibitors, like baxdrostat, seem to be a better alternative to MR inhibitors in treating conditions that are consequences of excessive aldosterone levels, as they do not affect the function of MR mediated by cortisol [82,106].

### 7.3. Adverse Effects

Baxdrostat has been observed to be a rather well-tolerated drug [101]. Reported side effects have been mild and mostly include headache, nasopharyngitis, asthenia, and diarrhea [99,100]. The BrigHTN trial had found no serious adverse events associated with baxdrostat [94]. During the trial, no adrenal insufficiency occurred, and only six cases of hyperkalemia were observed [94].

### 7.4. Interactions Between Baxdrostat and Other Medications

A study by Freeman et al. [107] has evaluated the effects of baxdrostat on the pharmacokinetics of metformin. Baxdrostat inhibits the multidrug and toxin extrusion 1 (MATE1) and MATE2-K renal transporters, and metformin is a substrate of MATE; therefore, the potential of possible interactions between the drugs was worrying [106,107,108]. Yet, Freeman et al. [107] found that baxdrostat and metformin were tolerated well when administered together. Moreover, the study has suggested that diabetic patients with hypertension who receive both baxdrostat and metformin are unlikely to need dose adjustment [107]. Furthermore, there is an ongoing phase 3 trial, NCT06268873, that will assess the efficacy of baxdrostat and dapagliflozin in patients with CKD and hypertension [109]. Studies are also underway to assess the effect of a strong CYP3A4 inhibitor (itraconazole) on the pharmacokinetics of baxdrostat in healthy volunteers. One study, titled “A Study to Investigate the Pharmacokinetics of Baxdrostat When Given Alone and in Combination with Itraconazole,” utilized an open-label, three-period sequential design consisting of baxdrostat administered alone, then itraconazole, and finally both drugs in combination. The study was completed in June 2024, but detailed results regarding pharmacokinetic interactions have not yet been published [110].

### 7.5. Possible Concerns About Baxdrostat

Issues have been raised regarding baxdrostat’s efficacy in patients with CYP11B2 genetic polymorphisms that vary across different ethnic groups, especially since the BrigHTN and HALO trials both included mostly Caucasian people [94,103]. Some studies such as Sydorchuk et al. [111] have found that the polymorphic site of CYP11B2 (rs1799998) gene can be linked to elevated blood pressure and higher levels of blood aldosterone, while others such as Byrd [96] et al. have not been able to find such associations. While there is no published evidence that baxdrostat may be less effective in patients with different CYP11B2 genetic polymorphisms yet, some ongoing clinical trials may provide more information about such concerns [112]. BaxHTN (NCT06034743) is a phase 3, multi-national, global trial with 263 locations all over the world that includes 796 patients with uncontrolled or resistant hypertension [104]. BaxAsia (NCT06344104) is a phase 3 clinical trial that includes 326 patients with uncontrolled or resistant hypertension primarily from Asia [113]. Recruiting patients of different ethnicities can help broaden the understanding of baxdrostat use not only in patients with possible CYP11B2 genetic polymorphisms but also takes into consideration issues like higher dietary salt intake and salt sensitivity which have been observed in people living in Asia [112].

### 7.6. Role of Baxdrostat in Specific Patient Populations

Clinical studies have shown that patients with resistant hypertension treated with baxdrostat experience significant reductions in both systolic blood pressure (SBP) and diastolic blood pressure (DBP) [94,114,115]. Even greater antihypertensive efficacy has been observed in patients with primary hyperaldosteronism [116]. Studies evaluating the potential beneficial effects of baxdrostat on the course of chronic kidney disease are also ongoing, although the results have not yet been published [117]. However, preliminary data on other aldosterone synthase inhibitors (ASIs) indicate a reduction in albuminuria and nephroprotective benefits in this group of patients [118].

## 8. Conclusions

Arterial hypertension, a key risk factor for cardiovascular disease, remains difficult to effectively control despite available diagnostic and therapeutic methods. Adrenal hormones—aldosterone and cortisol—play a significant role in its pathophysiology. Modulating them represents a promising treatment approach, particularly in resistant hypertension. Baxdrostat is a new, highly selective aldosterone synthase (CYP11B2) inhibitor that effectively lowers aldosterone levels without affecting cortisol synthesis. Clinical trials (including BrigHTN) have demonstrated significant reductions in systolic blood pressure in patients with resistant hypertension, with good tolerability and only mild side effects. The drug has a long half-life (approximately 29 h), allowing for once-daily dosing, and its efficacy and safety have also been confirmed in patients with impaired renal function. Numerous phase 3 trials are currently underway, including in Asian populations, to assess its role in the treatment of hypertension, chronic kidney disease, and primary hyperaldosteronism.

## Data Availability

The data used in this article were sourced from materials mentioned in the References section.
